# Human Host Defense Peptide LL-37 Stimulates Virulence Factor Production and Adaptive Resistance in *Pseudomonas aeruginosa*

**DOI:** 10.1371/journal.pone.0082240

**Published:** 2013-12-13

**Authors:** Nikola Strempel, Anke Neidig, Michael Nusser, Robert Geffers, Julien Vieillard, Olivier Lesouhaitier, Gerald Brenner-Weiss, Joerg Overhage

**Affiliations:** 1 Karlsruhe Institute of Technology (KIT), Institute of Functional Interfaces, Karlsruhe, Germany; 2 Helmholtz Center for Infection Research, Genome Analytics, Braunschweig, Germany; 3 UMR CNRS 6014 COBRA, University of Rouen, Evreux, France; 4 Laboratory of Microbiology Signals and Microenvironment LMSM EA 4312, University of Rouen, Evreux, France; UC Berkeley, United States of America

## Abstract

A multitude of different virulence factors as well as the ability to rapidly adapt to adverse environmental conditions are important features for the high pathogenicity of *Pseudomonas aeruginosa*. Both virulence and adaptive resistance are tightly controlled by a complex regulatory network and respond to external stimuli, such as host signals or antibiotic stress, in a highly specific manner. Here, we demonstrate that physiological concentrations of the human host defense peptide LL-37 promote virulence factor production as well as an adaptive resistance against fluoroquinolone and aminoglycoside antibiotics in *P. aeruginosa* PAO1. Microarray analyses of *P. aeruginosa* cells exposed to LL-37 revealed an upregulation of gene clusters involved in the production of quorum sensing molecules and secreted virulence factors (PQS, phenazine, hydrogen cyanide (HCN), elastase and rhamnolipids) and in lipopolysaccharide (LPS) modification as well as an induction of genes encoding multidrug efflux pumps MexCD-OprJ and MexGHI-OpmD. Accordingly, we detected significantly elevated levels of toxic metabolites and proteases in bacterial supernatants after LL-37 treatment. Pre-incubation of bacteria with LL-37 for 2 h led to a decreased susceptibility towards gentamicin and ciprofloxacin. Quantitative Realtime PCR results using a PAO1-*pqsE* mutant strain present evidence that the quinolone response protein and virulence regulator PqsE may be implicated in the regulation of the observed phenotype in response to LL-37. Further experiments with synthetic cationic antimicrobial peptides IDR-1018, 1037 and HHC-36 showed no induction of *pqsE* expression, suggesting a new role of PqsE as highly specific host stress sensor.

## Introduction

*Pseudomonas aeruginosa* is a widespread Gram-negative water and soil bacterium, which is, in addition, one of the most important opportunistic human pathogens causing severe infections in immunocompromised persons, such as burn wound, catheter and urinary tract infections or chronic pneumonia in cystic fibrosis (CF) patients [1]. Due to a large arsenal of intrinsic resistance mechanisms such as a low outer membrane permeability, the expression of antibiotic cleaving enzymes, and the existence of multidrug efflux pump systems, *P. aeruginosa* is inherently resistant to various commonly used antibiotics [2], [3].

Multiple virulence factors have been identified to affect the pathogenicity of *P. aeruginosa*. These factors comprise the expression of extracellular appendices flagella, type IV pili and type III secretion systems, the production of alginate and lipopolysaccharide (LPS) and the synthesis of secreted exocompounds such as proteases (e.g. elastase) and other enzymes, toxins, phenazines, rhamnolipids, hydrogen cyanide (HCN) and quorum sensing molecules (e.g. 4-quinolone PQS) [4], [5]. *P. aeruginosa* virulence is controlled by a highly complex and in large parts not fully understood signaling network including the Las, Rhl and PQS quorum sensing systems which induce the expression of various virulence factors e.g. in response to high cell densities or other external stimuli like iron limitation [6].

Adaptive resistance in *P. aeruginosa* has been reported for aminoglycosides and different cationic antibiotics such as polymyxins and the bovine cationic peptide indolicidin. Although the phenomena of adaptive resistance against the aminoglycoside gentamicin and the polypeptide antibiotic polymyxin B was first mentioned decades ago [7], [8], the underlying signaling pathways and involved defense mechanisms have been elucidated in parts in recent studies [9], [10], [11], [12], [13]. While the two-component systems PhoP-PhoQ and PmrA-PmrB recognize low Mg^2+^ concentrations and phosphate deprivation in the environment, ParR-ParS and CprR-CprS have been shown to directly sense cationic compounds, such as polymyxin B, colistin, indolicidin and, amongst others, the synthetic antimicrobial peptides HHC-36 and IDR-1018 [12], [13]. Activation of mentioned two-component systems induces the expression of the LPS modifying operon *arnBCADTEFugd*, leading to a reduced net charge of LPS due to the addition of 4-aminoarabinose to lipid A, which impairs the self-promoted uptake of cationic compounds across the outer membrane and thereby enhances tolerance to these compounds [4], [14].

The increasing occurrence of infections caused by multidrug-resistant bacteria, which tolerate even high concentrations of common antibiotics, calls for the rapid development and clinical application of new anti-infective strategies [15]. Host defense peptides, also termed as antimicrobial peptides (AMPs), have been considered as promising compounds to combat multi-resistant pathogens due to their combinatory actions as antimicrobial, antibiofilm and immunomodulatory agents [15]. The major human host defense peptide, LL-37, is the only cathelicidin class peptide produced in humans and exhibits a modest antibacterial activity against a variety of different pathogens including *Staphylococcus epidermidis*, *Staphylococcus aureus*, *Streptococcus pneumonia* and *P. aeruginosa* [16]. Additionally, it has been shown to prevent the formation of resistant biofilms and stimulate biofilm dispersal in various bacteria when applied at sublethal concentrations [17]. LL-37 is synthesized by phagocytes, epithelial cells and keratinocytes and has been detected in a large number of different cells, tissues and body fluids at varying concentrations [16]. During infectious diseases, immune cells and epithelial cells secrete a battery of host defense compounds, with either direct antimicrobial or immunomodulatory activities, including cationic peptides [5]. Extracellular LL-37 levels have been observed to be severely increased, reaching local concentrations of 15–20 µg/ml e.g. in the lung fluid of newborns suffering from pulmonary infections [18] and in cystic fibrosis patients [19] – diseases, which are often linked to *P. aeruginosa* infections [1].

Previous studies demonstrated an influence of human opioids [20], [21], natriuretic peptides [22], INF-γ [23] and the polypeptide antibiotic colistin [24] on virulence and quorum sensing in *P. aeruginosa*. However, this has not been investigated for human cationic host defense peptides so far. In this study, we elucidated the response of *P. aeruginosa* towards physiological concentrations of LL-37 by global transcriptional studies and metabolite analyses and observed a strong induction of virulence factor production as well as an increase in efflux pump expression during incubation with LL-37. Further experiments revealed an involvement of the quinolone signal response protein PqsE in the regulation of this LL-37 stimulated enhanced virulence factor production and adaptive resistance in *P. aeruginosa*.

## Materials and Methods

### Bacterial Strains, Media and Antimicrobial Peptides

Bacterial strains used in this study are listed in Table 1. Transposon mutants PAO1-*pqsE* and PA14-*mexH* were confirmed by PCR (data not shown). All experiments were performed in Mueller Hinton (MH) broth (Merck, Darmstadt, Germany). Bacteria were routinely grown at 37°C with shaking at 170 rpm. Antimicrobial peptides were kindly provided by Prof. Robert Hancock (University of British Columbia, Vancouver, Canada) or purchased from Anaspec (Fremont, CA, USA). The amino acid sequences of antimicrobial peptides used and their minimal inhibitory concentrations (MIC) against PAO1 WT are shown in Table 2. Peptide stock solutions of 2 mg/ml were prepared in sterile ultra pure DI water and stored at −20°C until needed.

**Table 1:** *P. aeruginosa* strains used in this study.

Strain	Description	Reference
**PAO1 WT**	H103 (PAO1 wild-type strain)	[61]
**PAO1-** ***pqsE***	Transposon insertion in *pqsE* (PA1000), Mutant ID PAO1_lux_76:C11, Tc^R^	[38]
**K767**	Wild-type of strains K1521, K1536, K1523, K1455, K1525, K2415, K2896	[57]
**K1521**	K767Δ*mexCD*	[57]
**K1536**	K767Δ*nfxB*	[57]
**K1523**	K767Δ*mexB*	[57]
**K1455**	K767Δ*nalB*	[62]
**K1525**	K767Δ*mexXY*	[63]
**K2415**	K767Δ*mexZ*	[63]
**K2896**	K767 Δ*mexB*Δ*mexCD*Δ*mexXY*	[57]
**K2153**	Clinical isolate, wild-type of efflux mutants K2892 and K2376	[64]
**K2892**	K2153Δ*mexF*	[64]
**K2376**	K2153Δ*mexS*	[64]
**PA14 WT**	PA14 wild-type strain	[65]
**PA14-** ***mexH***	Transposon insertion in *mexH* (PA4206), Mutant ID: PAMr_nr_mas_09_3:C7, Gm^R^	[65]

Gm^R^: gentamicin resistance, Tc^R^: tetracycline resistance.

**Table 2:** Antimicrobial peptides used in this study.

Peptide	Sequence	Source/Reference	MIC [µg/ml] against PAO1 WT^a^
**LL-37**	LLGDFFRKSKEKIFKEFKRIVQRIKDFLRNLVPRTES	[66]	16
**HHC-36**	KRWWKWWRR	[47]	16
**IDR-1018**	VRLIVAVRIWRR	[45]	16
**1037**	KRFRIRVRV	[46]	16

^a^ Minimal inhibitory concentrations (MIC) of PAO1 WT against different antimicrobial peptides were determined in MH broth using a standard two-fold serial dilution protocol for microtiter plates. Data represent mode MIC values of three independent experiments for each strain.

### MIC (Minimal Inhibitory Concentration) Determination

MIC values were determined using a standard broth microdilution protocol as described previously [25]. Growth in MH medium in presence or absence of antibiotics or antimicrobial peptides was monitored after 18 h of incubation at 37°C. In case of experiments with cationic peptides, 96-well polypropylene microtiter plates (Eppendorf, Hamburg, Germany) were used in order to prevent high MIC values due to the binding of cationic peptides to polystyrene. MIC values against antibiotics ciprofloxacin and gentamicin were determined in 96-well polystyrene microtiter plates (Nunc, Thermo Fisher Scientific, St. Leon-Rot, Germany).

### RNA Extraction, cDNA Synthesis and Microarray Analysis

For global gene expression studies, three independent mid-log phase cultures of *P. aeruginosa* PAO1 were challenged with LL-37 (20 µg/ml) for 2 h. Untreated bacterial cultures served as negative controls. To ensure homogenous gene expression profiles within treated and untreated groups enabling a precise analysis of transcriptional changes, we used bacteria from the exponential growth phase. Due to the higher cell number in this experiment (∼5×10^8^ cells/ml) in comparison to the MIC assay (5×10^5^ cells/ml), used LL-37 concentrations of 20 µg/ml did not affect bacterial growth during the incubation time. This was confirmed by measuring the optical density of bacterial cultures at 600 nm (OD_600_), resulting in comparable OD_600_ values in the range of 0.7–1.2 in treated samples and untreated controls after 2 h of incubation. Following peptide treatment, total RNA was extracted using RNA protect reagent and RNeasy Mini Kit (Qiagen, Hilden, Germany) according to the manufacturer’s instructions. Remaining contaminating DNA was removed from the samples in an off-column DNAse digestion procedure using Ambion® DNA-free™ Kit (Life Technologies GmbH, Darmstadt, Germany). RNA quantity and quality was checked photometrically.

First strand cDNA synthesis from 10 µg total RNA as well as cDNA fragmentation into 50–200 bp fragments, biotin-labeling and subsequent hybridization to *Affymetrix GeneChip DNA Microarrays Pae_G1a* was carried out according to the manufacturer’s standard protocol (Affymetrix UK Ltd, Freiburg, Germany). Each sample was hybridized to at least two microarray chips as technical repeat. Only genes which showed more than 1.5-fold changes in gene expression between LL-37-treated bacteria and untreated controls were included in further analyses.

### Quantitative Real Time PCR (qRT-PCR)

Quantitative Realtime PCR (qRT-PCR) experiments were performed in order to verify microarray results of specific dysregulated genes. To this aim, *P. aeruginosa* cultures were grown until mid-exponential phase following incubation with peptides LL-37, IDR-1018, 1037 or HHC-36 (20 µg/ml each; MIC values: 16 µg/ml (Table 2)) for 2 h as described for microarray analysis. Isolation of total RNA was carried out using RNA protect reagent and the RNeasy Mini Kit (Qiagen, Hilden, Germany) according to the manufacturer’s instructions. Remaining contaminating DNA was removed from the samples in an off-column DNAse digestion procedure using Ambion® DNA-free™ Kit (Life Technologies GmbH, Darmstadt, Germany). RNA quantity and quality was checked photometrically. RNA was converted into first strand cDNA using random hexamers and Maxima Reverse Transcriptase (Thermo Fisher Scientific, St. Leon-Rot, Germany) in a standard PCR protocol which was provided by the manufacturer. cDNA was diluted to a concentration of 4 ng/µl and directly utilized as template for qRT-PCR reactions using the KAPA SYBR Fast Universal qPCR MasterMix (Peqlab Biotechnologie GmbH, Erlangen, Germany) in an Abi 7300 Real Time PCR System (Applied Biosystems Deutschland, Darmstadt, Germany) as described previously [26]. Analysis of melting curves of PCR products ensured specificity of the PCR reactions. Primers used for determination of *P. aeruginosa* gene expression were designed with *Primer Express* (Applied Biosystems Deutschland, Darmstadt, Germany). Primer sequences are shown in Table S1. Obtained ct-values were normalized to the expression of housekeeping gene *rpoD* which was not affected by LL-37 treatment as shown by microarray analysis. Samples were assayed at least three times in duplicate (n≥6).

### Time-killing Curves

PAO1 WT cultures were grown to mid-log phase and then incubated with LL-37 (20 µg/ml) or without peptide as controls for 2 h at 37°C in MH broth. Following dilution of bacterial cultures to 10^7^ cells/ml in MH broth, antibiotics ciprofloxacin (0.18 µg/ml) or gentamicin (1.5 µg/ml) were added at 3-fold MIC concentrations. Samples were serially diluted and plated out on LB agar after 0, 2, 5, 7, 10, 15, 20, 30, 60 and 90 min of incubation using an optimized drop plate method [27]. Experiments were conducted in triplicates, each with an independent bacterial culture.

### Measurement of Pyocyanin and Elastase Activity

For detection and quantification of virulence-associated metabolites and enzymes, PAO1 WT mid-log phase cultures were incubated with 20 µg/ml LL-37 for 21 h. Untreated cultures served as negative controls. Following peptide treatment, bacteria were spun down by centrifugation (30 min, 9000×*g*, 4°C) and the supernatants were passed through a 0.22 µm syringe filter (Sarstedt, Nümbrecht, Germany).

Pyocyanin was extracted from 2 ml supernatant by adding 2 ml chloroform following re-extraction with 2 ml 0.2 M HCl and subsequent measurement of absorption at 520 nm (A_520_) as described previously [28]. A_520_ was normalized against OD_600_ of the bacterial culture.

Elastase activity in supernatants was determined by an elastin congo red (ECR) assay which was previously described by Pearson *et al.* [29]. Elastolytic levels were normalized against the cell density of each sample (OD_600_).

Pyocyanin and elastase activity analyses were performed at least with six independent bacterial cultures. All data was statistically analyzed using the non-parametric Mann-Whitney test.

### PQS Measurement by Liquid Chromatography/Tandem Mass Spectrometry (LC-MS/MS)

Supernatants of PAO1 WT bacteria grown for 21 h in the presence or absence of LL-37 were obtained as described above for pyocyanin and elastase analyses. Supernatants were mixed with 2 volumes dichloromethane and vortexed for 1 min. After centrifugation (10 min, 9000×*g*, 4°C), the lower organic layer containing PQS was transferred into a new reaction tube following evaporation under nitrogen gas at room temperature. The pellet was then resuspended in pure methanol following quantification by LC-MS/MS. To this aim, a quaternary HPLC pump and an autosampler of the series 200 from Perkin Elmer (Überlingen, Germany) were used. The protocol was adapted from two methods described previously [30], [31] with the following modifications. The separation was performed on a Zorbax Eclipse XCB-C8 5 µm, 150×3.6 mm HPLC column (Agilent, USA). The mobile phase consisted of acetonitrile - water 80∶20 (v/v) with 100 µM EDTA and 0.1% acetic acid at a flow rate of 0.40 ml/min. The injection volume was set to 10 µl per sample. Electro-Spray-Ionisation (ESI)-MS was performed on an API 365 triple quadrupole mass spectrometer (PE Sciex, Toronto, Canada) using a turbo ion spray interface used in *positive mode*. Single MS experiments (Q1 scan), MS/MS experiments (product ion scan, PIC) and multiple reaction monitoring (MRM) were performed using nitrogen as curtain gas, nebulizer gas, heater gas and collision gas. Instrumental parameters were optimized by infusion experiments with PQS standard solution (10 µg/mL; Sigma-Aldrich, Seelze, Germany) infused into the mass spectrometer using a syringe pump (Harvard Apparatus Inc. South Natick, MA, USA) at a flow rate of 10 µl/min. To quantify PQS with high selectivity and sensitivity, MRM experiments were performed using the transitions from precursor ion to fragment ion: 260/175 (quantifier), 260/146, 260/147 and 260/188 (qualifier). An external calibration was performed using PQS standard solutions with concentrations ranging from 10 ng/ml up to 1000 ng/ml.

PQS concentrations were normalized against the cell density of each sample (OD_600_). Analyses were performed with six independent bacterial cultures and data was statistically analyzed using the non-parametric Mann-Whitney test.

### HCN/CN^−^ Quantification

In order to determine levels of toxic HCN in response to LL-37, *P. aeruginosa* cultures were grown until mid-log phase followed by 2 h of incubation with LL-37 (20 µg/ml) or without peptide as negative control. Subsequently, cells were sedimented by centrifugation (30 min, 9000×*g*, 4°C) and supernatants were passed through a 0.22 µm syringe filter (Sarstedt, Germany). Quantification of HCN/CN^−^ production was carried out using a polarographic approach delevoped by Blier *et al.* [32]. Since HCN/CN^−^ production in *P. aeruginosa* mainly occurs during the exponential growth phase with a peak after 5 h post-inoculation [32], samples for HCN quantification were taken 2 h after LL-37 addition, which corresponds to the late log phase of bacterial growth. Mean values and pooled standard deviations were calculated from three independent experiments, each measured in triplicate, and normalized to OD_600_ values. Statistical significance was verified by a two-sided t-test for independent samples.

### Microarray Data Accession Number

Complete microarray data is deposited in ArrayExpress under accession number E-MEXP-3970.

## Results

### LL-37 Induces the Expression of Virulence Factor Synthesis and Multidrug Efflux Pump Genes in *P. aeruginosa* PAO1

In order to get a detailed insight into the global response of *P. aeruginosa* to physiological concentrations of the human host defense peptide LL-37, we performed gene expression studies using DNA microarray technology. To this aim, mid-log phase *P. aeruginosa* cells were exposed to 20 µg/ml LL-37 for 2 h following RNA extraction and transcriptional analysis. Untreated cultures served as negative controls. Determined OD_600_ values were comparable in treated *P. aeruginosa* samples and control cultures, ranging between 0.7 and 1.2 after the incubation time of 2 h, which confirmed that growth was not inhibited by applied LL-37 concentrations. Additional CFU counts showed furthermore that the relation between OD_600_ and cell counts was not altered by the applied peptide concentrations. An OD_600_-value of 1.0 corresponded to 0.8±0.2×10^9^ CFU/ml in control cultures and to 1.1±0.3×10^9^ CFU/ml in LL-37-treated samples after 2 h of incubation.

**Figure 1:**
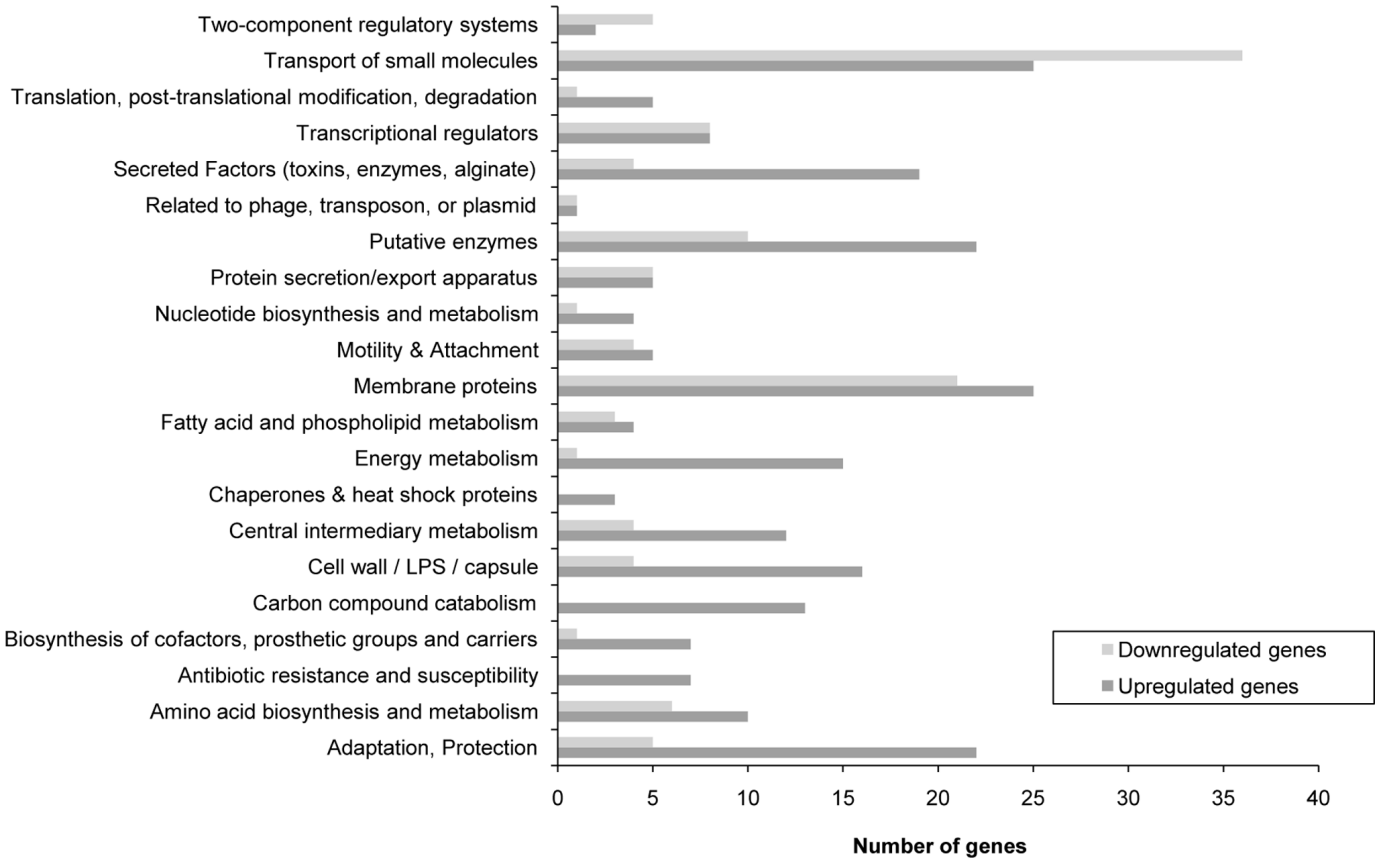
Summarized microarray data of dysregulated *P. aeruginosa* genes in response to LL-37. Mid-log phase cultures of *P. aeruginosa* PAO1 were grown in MH broth containing either 20 µg/ml LL-37 or no LL-37 for 2 h at 37°C following RNA extraction and microarray analysis. The graph shows functions of more than 1.5-fold up- or downregulated genes according to the *Pseudomonas* Genome Database [28]. Hypothetical genes are not shown.

Regarding microarray results, comparison of LL-37-treated bacteria with untreated controls revealed a total number of 420 dysregulated genes (cut-off: 1.5-fold up- or downregulation), of which 280 genes were upregulated and 140 genes were downregulated (Tables 3, S2 and S3). Figure 1 summarizes the functions of dysregulated genes in response to LL-37 and illustrates the diversity of *P. aeruginosa* stress response to LL-37, since 21 out of the 25 defined gene function classes [33] were affected by the cationic peptide. Quantitative RT-PCR experiments on selected genes were performed in order to confirm microarray data (Table 4).

**Table 3:** Microarray results of selected dysregulated genes of *P. aeruginosa* PAO1 WT in response to 2 h of incubation with LL-37 (20 µg/ml) compared to untreated controls.

PA number	Gene name	Gene product	Fold change in gene expression
PQS biosynthesis and response (quorum sensing, virulence factor)			
PA0996	*pqsA*	Probable coenzyme A ligase	+2.1
PA0997	*pqsB*	PqsB	+1.9
PA0998	*pqsC*	PqsC	+1.6
PA0999	*pqsD*	3-oxoacyl-[acyl-carrier-protein] synthase III	+1.7
PA1000	*pqsE*	Quinolone signal response protein	+2.1
Pyocyanin biosynthesis (virulence factor)			
PA0051	*phzH*	Potential phenazine-modifying enzyme	+1.6
PA1001	*phnA*	Phenazine biosynthesis protein PhnA	+2.0
PA1002	*phnB*	Phenazine biosynthesis protein PhnB	+2.2
PA1901	*phzC2*	Phenazine biosynthesis protein PhzC	+4.7
PA1902	*phzD2*	Phenazine biosynthesis protein PhzD	+5.8
PA1903	*phzE2*	Phenazine biosynthesis protein PhzE	+6.1
PA1904	*phzF2*	Probable phenazine biosynthesis protein	+6.0
PA1905	*phzG2*	Probable pyridoxamine 5′-phosphate oxidase	+6.3
PA4209	*phzM*	Probable phenazine-specific methyltransferase	+5.3
PA4210	*phzA1*	Probable phenazine biosynthesis protein	+7.8
PA4211	*phzB1*	Probable phenazine biosynthesis protein	+4.5
PA4217	*phzS*	Flavin-containing monooxygenase	+5.3
Elastase biosynthesis (virulence factor)			
PA1871	*lasA*	LasA protease precursor	+1.8
PA3724	*lasB*	Elastase LasB	+2.1
Hydrogen cyanide (HCN) production (virulence factor)			
PA2193	*hcnA*	Hydrogen cyanide synthase HcnA	+2.4
PA2194	*hcnB*	Hydrogen cyanide synthase HcnB	+2.6
PA2195	*hcnC*	Hydrogen cyanide synthase HcnC	+2.1
Rhamnolipid production			
PA1130	*rhlC*	Rhamnosyltransferase 2	+2.2
PA3478	*rhlB*	Rhamnosyltransferase chain B	+2.0
PA3479	*rhlA*	Rhamnosyltransferase chain A	+1.5
Porins, efflux pumps			
PA3279	*oprP*	Phosphate-specific outer membrane porin OprP precursor	−5.5
PA3280	*oprO*	Pyrophosphate-specific outer membrane porin OprO precursor	−8.2
PA4205	*mexG*	Hypothetical protein	+10.2
PA4206	*mexH*	Probable RND efflux membrane fusion protein precursor	+4.9
PA4207	*mexI*	Probable RND efflux transporter	+2.5
PA4208	*opmD*	Probable outer membrane protein precursor	+3.1
PA4597	*oprJ*	Multidrug efflux outer membrane protein OprJ precursor	+3.7
PA4598	*mexD*	RND multidrug efflux transporter MexD	+4.4
PA4599	*mexC*	RND multidrug efflux membrane fusion protein MexC precursor	+9.1
PA4600	*nfxB*	Transcriptional regulator NfxB	+1.9
Lipopolysaccharide (LPS) modification			
PA3552	*arnB*	ArnB	+1.6
PA3553	*arnC*	ArnC	+1.6
PA3555	*arnD*	ArnD	+1.5
PA3556	*arnT*	Inner membrane L-Ara4N transferase ArnT	+2.0
PA3557	*arnE*	ArnE	+2.0
PA3558	*arnF*	ArnF	+2.2
PA3559	*ugd*	Probable nucleotide sugar dehydrogenase	+2.8
Two-component system PmrA-PmrB			
PA4773		Hypothetical protein	+4.9
PA4774		Hypothetical protein	+3.2
PA4775		Hypothetical protein	+2.2
PA4776	*pmrA*	Two-component regulator system response regulator PmrA	+1.9

**Table 4:** qRT-PCR analysis of *P. aeruginosa* PAO1 WT and PAO1-*pqsE* mutant gene expression in response to LL-37 (20 µg/ml)^a^.

Gene	PAO1 WT	PAO1-*pqsE*
PA4598 (*mexD*)	1.8±0.1^b^	1.9±0.2
PA4206 (*mexH*)	7.5±0.4	1.0±0.1
PA1000 (*pqsE*)	1.7±0.3	n.d.
PA3724 (*lasB*)	2.6±0.5	0.8±0.2
PA2194 (*hcnB*)	1.8±0.3	0.8±0.1
PA1901 (*phzC2)*	2.8±0.4	1.0±0.3
PA4776 *(pmrA)*	1.6±0.2	0.9±0.2
PA3556 *(arnT)*	1.5±0.2	1.8±0.2

*P. aeruginosa* PAO1 WT or PAO1-*pqsE* were grown in MH broth containing either 20 µg/ml LL-37 or no LL-37 (control) for 2 h at 37°C following RNA isolation and qRT-PCR analysis.^a^ Mid-log phase cultures of

≥6). ct values were normalized against expression of housekeeping gene *rpoD.* Fold changes in gene expression of LL-37-treated cells compared to untreated controls were calculated using the ΔΔct method [67]. n.d.: not determined.^b^ Mean averages and standard deviations of three independent experiment, each analyzed at least in duplicate (n

Most strikingly, the microarray data showed an upregulation by 2–8-fold of gene clusters involved in quorum sensing molecule and virulence factor synthesis. Among these were genes coding for the *Pseudomonas* quinolone signal (PQS) – *pqsABCD* (PA0996–0999) - and the production of secreted toxic metabolites phenazine (PA0051, PA1001/1002, PA1901–1905, PA4209–4211, PA4217), HCN (PA2193–2195), elastase (PA1871, PA3724) and rhamnolipids (PA3478/3479, PA1130) (Table 3). *PqsE* (PA1000), which is involved in the regulation of virulence factor expression, influencing e.g. pyocyanin, rhamnolipid and HCN production [6], was also 2-fold upregulated during LL-37 incubation. Although expression of rhamnolipid biosynthesis genes *rhlA* (PA3479), *rhlB* (PA3478) and *rhlC* (PA1130) was enhanced by LL-37 contact, the two main regulators of rhamnolipid production, *rhlI* (PA3476) and *rhlR* (PA3477) [34] were not induced (see Tables S2 and S3). Additional qRT-PCR experiments indicated rather a downregulation of major quorum sensing regulators *rhlR* (fold change −2.0±0.3) and *lasR* (fold change −1.9±0.5) in response to LL-37. In general, only a few genes (in total 9) encoding transcriptional regulators were more than 1.5-fold upregulated by LL-37 (see Table S2).

In accordance with previous studies [9], [12], we observed an induction of the *arnBCADTEFugd* LPS modification operon (PA3552-3559) and the two-component regulator *pmrA* (PA4776). In addition, our microarray data demonstrated an upregulation of Resistance Nodulation Division (RND) efflux pumps genes *mexCD-oprJ* (PA4597-4599) and *mexGHI-opmD* (PA4205-4208), which are also involved in multidrug resistance of *P. aeruginosa* by exporting antibiotics and other toxic compounds [3], [35], [36], [37], whereas genes encoding porin proteins were downregulated by LL-37 (Table 3).

### Susceptibility of Different *P. aeruginosa* Efflux Mutant Strains to LL-37

Since we observed an upregulation of two RND efflux pump systems in response to LL-37, we performed LL-37 susceptibility tests with respective efflux pump mutants PA14-*mexH* and K1521 (K767Δ*mexCD-oprJ*) in comparison to the corresponding wild-type strains PA14 and K767. Additional experiments included efflux mutants K1523 (K767*ΔmexB*), K1525 (K767*ΔmexXY*) and K2892 *(*K2153*ΔmexF)* and K2153, the wild-type strain of K2892. All tested efflux mutants and corresponding wild-type strains showed identical MIC values for LL-37 (16 µg/ml for PA14-*mexH*, K1521, K1523, K1525 and wild-types PA14 WT and K767; 32 µg/ml for K2892 and wild-type K2153), indicating no impact of a single pump knockout of either MexAB-OprM, MexCD-OprJ, MexXY-OprM, MexEF-OprN or MexGHI-OpmD on susceptibility to LL-37. Since export of many antibiotics is not restricted to one individual efflux pump [3], in case of a single pump knockout, other efflux systems could eventually take over functions of missing efflux pumps, resulting in unaffected MIC values. To test whether a multiple knockout of main *P. aeruginosa* RND efflux pumps MexAB-OprM, MexXY-OprM and MexCD-OprJ affects susceptibility to LL-37, MIC values were determined for triple mutant K2896 (K767Δ*mexB*Δ*mexCD*Δ*mexXY*) and wild-type K767, both exhibiting identical MIC values of 16 µg/ml. Next, we could show that susceptibility to LL-37 was not influenced by overexpression of efflux pumps MexAB-OprM (K1455), MexCD-OprJ (K1536), MexXY-OprM (K2415) or MexEF-OprN (K2376) which also resulted in equal MIC values compared to corresponding wild-types K767 (16 µg/ml) or K2153 (32 µg/ml).

In summary, we conclude that susceptibility of *P. aeruginosa* PAO1 to LL-37 is independent of the tested efflux systems, although expression of RND efflux pump MexCD-OprJ was upregulated in response to LL-37 in our gene expression studies.

### LL-37 Enhances Antibiotic Resistance of *P. aeruginosa* PAO1 Towards Fluoroquinolone and Aminoglycoside Antibiotics

**Figure 2:**
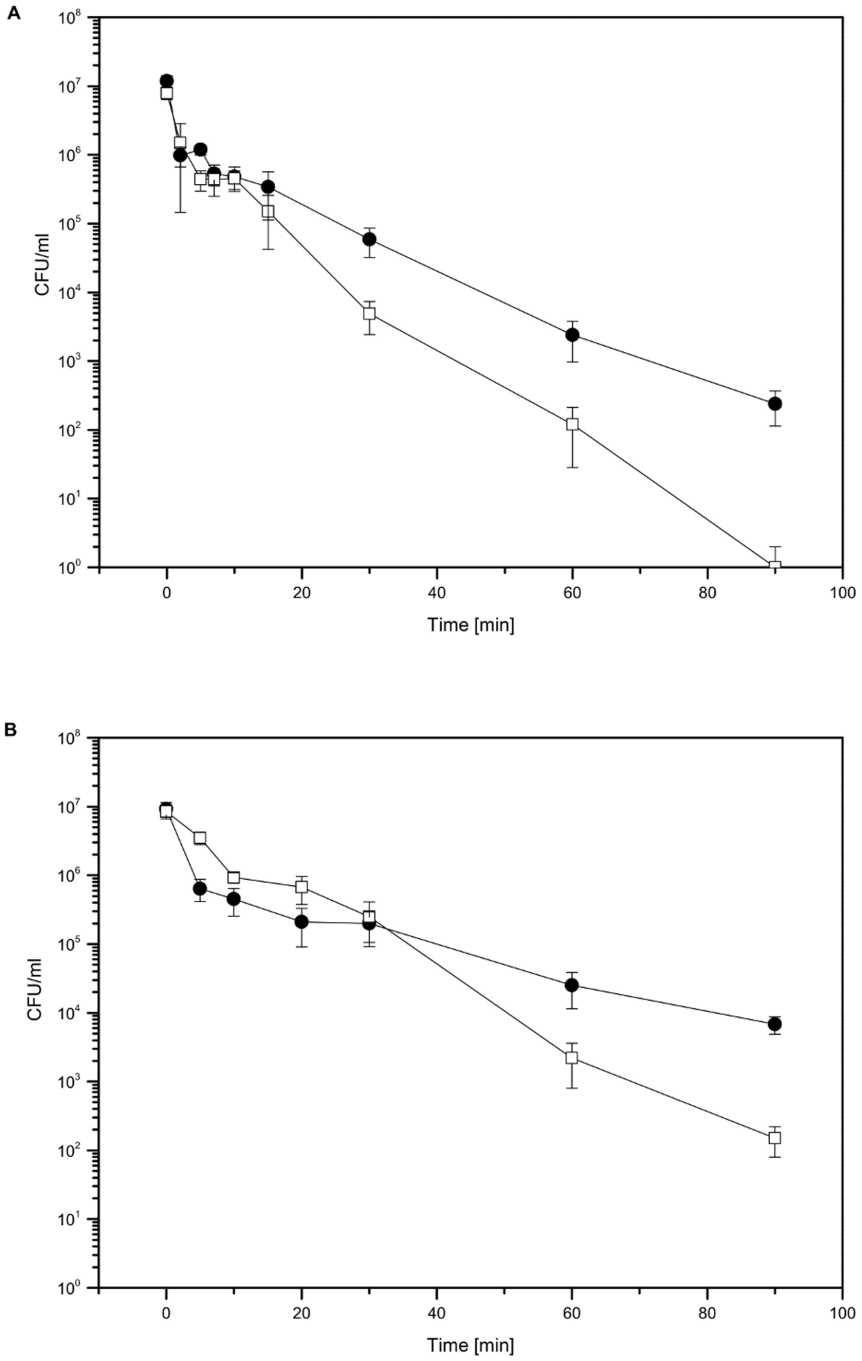
Time-killing of *P. aeruginosa* PAO1 by antibiotics ciprofloxacin (A) or gentamicin (B) in the absence or presence of LL-37. Mid-log phase bacterial cultures were incubated with either 20 µg/ml LL-37 (filled circles) or without LL-37 (open squares) for 2 h. Following dilution of bacterial cultures to 10^7^ cells/ml and addition of 3-fold MIC concentrations of antibiotics ciprofloxacin (0.18 µg/ml) or gentamicin (1.5 µg/ml), colony forming units at indicated time points were determined using the optimized drop plate method [27]. Experiments were performed in triplicate. The figure shows representative results of one experiment. Error bars indicate standard deviations of 10 spots per sample plated out on two different agar plates (n = 10).

To investigate whether LL-37 was able to trigger adaptive resistance mechanisms in *P. aeruginosa* towards different antibiotics, time dependent killing of *P. aeruginosa* PAO1 WT by the fluoroquinolone ciprofloxacin and the aminoglycoside gentamicin was monitored after a 2 h pre-incubation period with LL-37. The peptide itself did not affect bacterial growth as confirmed by determination of OD_600_. Colony forming units (CFUs) were counted after indicated time points during incubation with antibiotics and compared to cell numbers of control cultures without LL-37 pre-incubation. For ciprofloxacin, we observed an increased resistance of LL-37-treated bacteria compared to the untreated *P. aeruginosa* cultures already after 30 min of incubation. At this time point, CFU counts revealed only a 2-fold log reduction for LL-37-treated bacteria, but a more than 3-fold log reduction for controls without peptide treatment. After 90 min of incubation, no surviving bacteria in the control group could be detected, whereas *P. aeruginosa* cultures, which were pre-grown with LL-37, still showed cell numbers of approximately 220 CFU/ml (Figure 2A). Similar results were obtained with gentamicin, however, killing of bacteria was slower and less efficient compared to ciprofloxacin. During the first 30 min of incubation, both LL-37-treated and control bacteria showed a comparable 2-fold log reduction of culturable cells. CFU counts after 60 min demonstrated a beginning resistance of LL-37-treated cells (2×10^4^ CFU/ml) compared to untreated controls (2×10^3^ CFU/ml). After 90 min control cultures contained only 150 CFU/ml, whereas pre-incubation with LL-37 significantly increased cell numbers up to 6800 CFU/ml (Figure 2B). Statistical significance of differences between LL-37-treated bacteria and untreated controls at the end point of the experiment after 90 min of incubation with antibiotics was confirmed by a two-sided t-test for independent samples (p-value <0.001 for both antibiotics). Taken together, we could show that *P. aeruginosa* resistance to both fluoroquinolone and aminoglycoside antibiotics was enhanced by pre-incubation with the human cathelicidin LL-37.

### Virulence-associated Metabolite Production is Increased during LL-37 Treatment

As shown by our microarray analyses, LL-37 treatment of *P. aeruginosa* PAO1 induced the expression of several gene clusters which are known to be involved in the production of virulence-associated metabolites such as *las*, *pqs*, *phz* and *hcn* genes (Table 3).

**Figure 3:**
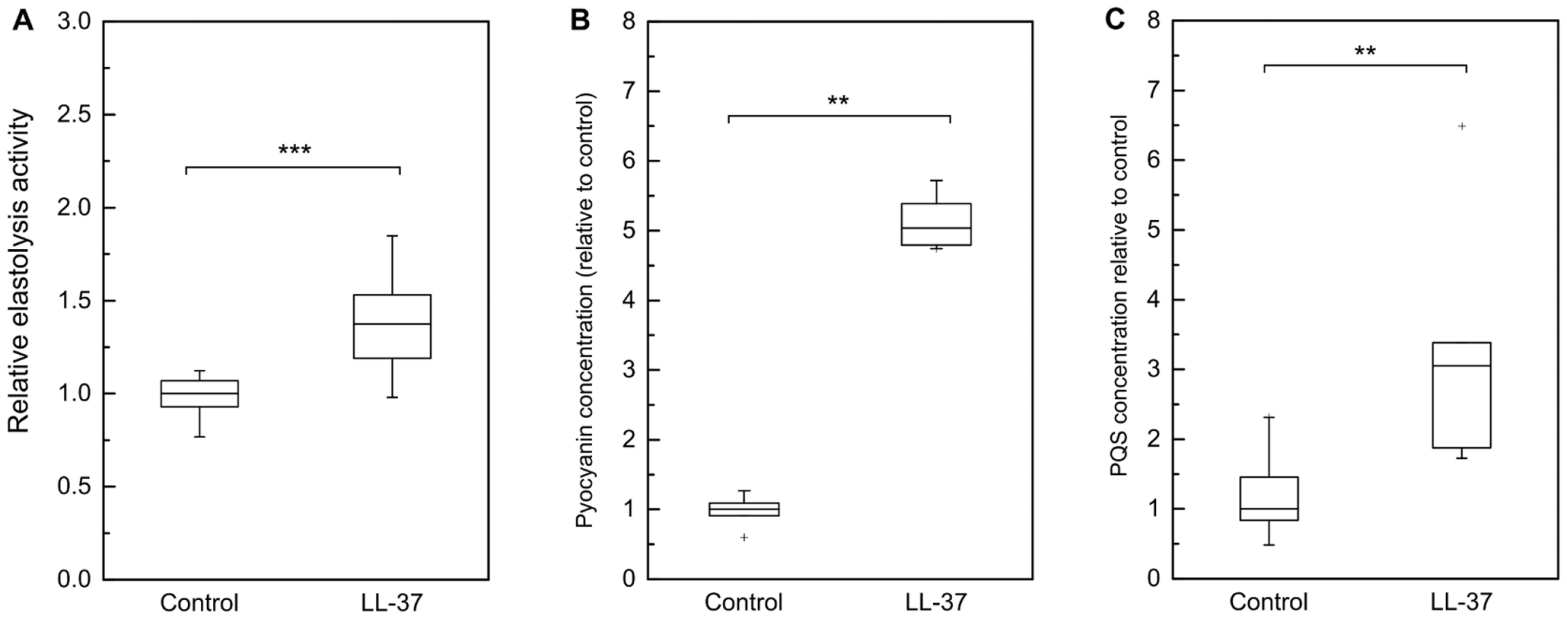
Quantification of metabolites elastase (A), pyocyanin (B) and PQS (C) in PAO1 WT supernatants after 21 h incubation without or with LL-37. Mid-log phase cultures of PAO1 WT were grown in MH broth containing either 20 µg/ml LL-37 or no LL-37 (control) for 21 h at 37°C. OD_600_ values after 21 h were comparable in treated samples and controls, indicating no growth inhibition by LL-37. Elastase activity (A) and pyocyanin concentration (B) in bacterial supernatants were determined photometrically. PQS levels (C) were quantified by LC-MS/MS. Boxes include median (black line), 25^th^ and 75^th^ percentiles of normalized data (n≥6). Statistical significance was calculated by Mann-Whitney-Test (**: p≤0.01, ***: p≤0.001).

To examine whether this upregulation of gene expression directly leads to an enhanced secretion of virulence factors, levels of pyocyanin, PQS, elastase and HCN were quantified in the bacterial supernatant. For elastase, pyocyanin and PQS determination, supernatants were analyzed after 21 h of incubation with LL-37 in order to ensure an accumulation of adequate amounts of metabolites for subsequent measurements. OD_600_ values after 21 h of incubation were only marginally decreased in LL-37-treated cultures compared to control cultures (p = 0.14; difference not statistically significant) and therefore LL-37 independent effects of divergent cell densities on quorum sensing and virulence factor levels could be excluded. Photometric determination of elastase expression and pyocyanin synthesis revealed significantly increased elastase (+1.4-fold) and pyocyanin (+5-fold) levels during LL-37 incubation compared to untreated controls (Figure 3A and 3B). Moreover, the LL-37-treated bacterial cultures in contrast to control cultures appeared intensely green (Figure S1), which was most likely due to the elevated levels of the green-blue fluorophore pyocyanin [6]. PQS content in bacterial supernatants was measured using LC-MS/MS and showed 3-fold higher levels in response to LL-37 as well (Figure 3C). HCN quantification also demonstrated increased HCN/CN^−^ levels in the supernatants of LL-37-treated bacteria compared to the untreated controls (Table 5). In conclusion, we could show, that the cathelicidin LL-37 not only affected the expression of various genes which are involved in quorum sensing cascades and virulence phenotype of *P. aeruginosa*, but was also able to directly enhance the secretion of toxic metabolites pyocyanin, elastase, PQS and HCN.

**Table 5:** HCN/CN^−^ concentrations in PAO1 WT supernatants^a^.

Sample	HCN/CN^−^ [µg/l]^b^
PAO1+ LL-37	899±31
PAO1 control	475±18

µg/ml LL-37 or no LL-37 (control) for 2 h at 37°C. Cell densities after 2 h peptide treatment were comparable in treated samples and controls, indicating no growth inhibition by LL-37. Supernatants were prepared by centrifugation following polarographic determination of HCN/CN^−^ content.^a^ Mid-log phase cultures of PAO1 WT were grown in MH broth containing either 20

= 9). Statistical significance of differences between mean values was confirmed by a two-sided t-test for independent samples (p<0.001).^b^ Mean averages and pooled standard deviations of three experiments, each measured in triplicate (n

### Involvement of *pqsE* in the Response of *P. aeruginosa* PAO1 to LL-37

Microarray analysis of *P. aeruginosa* PAO1 cells treated with LL-37 (20 µg/ml) indicated a dysregulation of 20 genes, of which 9 genes were more than 1.5-fold upregulated, whose gene products exhibit potential roles as transcriptional regulators (see Tables S2 and S3). In addition, we observed an increased expression of virulence regulator PA1000 (*pqsE*) (Table 3), the fifth gene of the PQS biosynthesis operon *pqsABCDE*. In order to analyze whether the increased biosynthesis of secreted virulence factors and the enhanced expression of efflux pumps MexCD-OprJ and MexGHI-OpmD in response to LL-37 was influenced by *pqsE* expression, we performed qRT-PCR experiments using a *pqsE* transposon insertion mutant (Mutant ID PAO1_lux_76:C11) of the PAO1mini-Tn*5 lux* transposon mutant library [38], which was either grown for 2 h in the presence or absence of LL-37. Whereas expression of efflux pump gene *mexD* and one gene of the LPS modification operon, *arnT*, still remained induced in response to LL-37 in the PAO1-*pqsE* mutant, our results demonstrated no alterations in the expression of genes *mexH*, *hcnB*, *lasB* and *phzC* in the PAO1-*pqsE* mutant treated with LL-37 compared to untreated PAO1*-pqsE* (Table 4). These findings suggest an involvement of *pqsE* in the regulation of our observed LL-37 induced adaptive resistance and virulence factor production in *P. aeruginosa.* To further investigate whether *pqsE* induction could represent a general response of *P. aeruginosa* to cationic peptides, we quantified *pqsE* expression after 2 h of incubation with the synthetic peptides IDR-1018, 1037 and HHC-36 (20 µg/ml each), but observed no changes in *pqsE* transcriptional levels in case of IDR-1018 (fold change +1.2±0.1) or rather a downregulation in case of 1037 (fold change −2.7±0.7) and HHC-36 (fold change: −3.0±0.7) in comparison to untreated bacteria.

## Discussion

The notable repertoire of virulence factors and the ability to rapidly develop adaptive resistances against antibiotics are two crucial factors for the great success of *P. aeruginosa* as an opportunistic human pathogen [2], [6]. Here we demonstrate that both, virulence factor production as well as the adaptive resistance against fluoroquinolone and aminoglycoside antibiotics, are considerably stimulated by the host defense peptide LL-37, when applied at concentrations that are comparable to the high LL-37 levels found in body fluids at sites of inflammation. Microarray data of LL-37-treated *P. aeruginosa* cells revealed an upregulation of quorum sensing genes *pqsABCDE* and significantly increased PQS levels in bacterial supernatants. PQS functions as a signaling molecule in cell-to-cell communication of *P. aeruginosa* and affects various cellular processes such as virulence, biofilm formation, swarming motility, antibiotic susceptibility and iron binding in an autoinduction mechanism which is dependent on a threshold concentration of PQS [6]. Since cell densities of LL-37-treated cultures and untreated controls were comparable after 2 h as well as after 21 h of incubation, growth effects as a factor influencing the level of quorum sensing signaling molecules and virulence factor production could be ruled out.

In contrast to PAO1 WT, expression of virulence factor genes and of efflux operon *mexGHI-opmD* was not enhanced in the PAO1-*pqsE* mutant during LL-37 incubation. These results indicate a regulatory function of *pqsE* in the adaptation to LL-37, which is comparable to the response to human peptide neuromodulator dynorphin [21] and its synthetic equivalent U50,488 in *P. aeruginosa* [20]. *PqsE* (PA1000), although located in one operon together with *pqsABCD*, is not implicated in PQS biosynthesis. Instead, it has been shown to influence the expression of more than 600 different genes, thus controlling e.g. the production of virulence factors phenazine, rhamnolipids, elastase and HCN and is required for full virulence of *P. aeruginosa* in mice [39], [40]. Although the recently solved crystal structure of PqsE and amino acid sequence analyses predict a hydrolase activity, there is still a controversy in the literature concerning the precise protein function [41]. Several studies showed that the inducing effect of PqsE on phenazine biosynthesis is controlled by the transcriptional regulator PqsR (MvfR) [40], [42], [43], whereas Farrow *et al.* observed a RhlR dependent stimulation of virulence factor production by PqsE also in the absence of PqsR [44]. Interestingly, our microarray analysis indicated no induction of major quorum sensing regulators *lasR, lasI, rhlI, rhlR* or *mvfR*. In accord with this, these genes were either unaffected or downregulated by U50,488 and the described induction of virulence and adaptive resistance genes was proposed to be regulated by *pqsE* alone in a yet unknown mechanism [20]. Cummins *et al.* reported an enhanced expression of *pqsB, pqsE, phzF and rhlB* in *P. aeruginosa* PAO1, in conjunction with an increased virulence against *Lactobacillus rhamnosus* in response to sublethal concentrations of the cationic antibiotic colistin [24].

In the present study, only LL-37, but none of the synthetic cationic peptides IDR-1018, 1037 and HHC-36 were able to induce *pqsE* expression, although they all target the outer cell membrane of Gram-negative bacteria in order to evolve their antibacterial actions [9]. Hence, the activation of *pqsE* expression and downstream effects appear to be dependent on other factors such as peptide structure or chemical properties. IDR-1018, 1037 and HHC-36 are small synthetic, 9 to 12 amino acid containing cationic peptides, based on the linear peptide Bac2A [45], [46], [47]. Studies on IDR-1018 structure revealed a β-turn conformation [45], whereas the 37 residue peptide LL-37 forms an α-helix during interaction with lipid bilayers [48]. In contrast to these linear peptides, polypeptide colistin (polymyxin E) exhibits a cyclic structure [24]. One main difference between *pqsE* affecting agents (LL-37, colistin, dynorphin and U50,488) and the synthetic peptides which showed no influence on *pqsE* expression (IDR-1018, 1037 and HHC-36), is the molecular mass of the agents, but whether this attribute is critical for the demonstrated induction of *pqsE* signal pathways, requires further investigation.

Moreover, our microarray data revealed an increased expression of the efflux operon *mexGHI-opmD*, which is implicated in the resistance against antibiotics norfloxacin [49] and vanadium [36]. In addition, *mexGHI-opmD* functions as a regulator of PQS and AHL synthesis and promotes quorum sensing and virulence in *P. aeruginosa*, presumably by exporting toxic quinolone intermediates [37]. Conversely, *mexGHI-opmD* expression is under the control of PQS and PqsE [39], [50] and has been recently shown to be directly regulated by pyocyanin in a SoxR dependent pathway [51]. Interestingly, only phenazine and PQS gene expression, but not the expression of transcriptional regulator SoxR was upregulated in response to LL-37 in our microarrays, suggesting the involvement of an alternative regulator in the observed induction of *mexGHI-opmD.* In contrast to our results, Cummins *et al*. [24] did not observe an upregulation of *mexGHI-opmD* operon by colistin - although *pqsE* and *phzF* expression was induced - emphasizing, again, the complex regulation of virulence and adaptive resistance in *P. aeruginosa* in response to different environmental stimuli.

Additionally to the enhanced production of toxic, virulence-associated compounds, we noticed an adaptive resistance of *P. aeruginosa* against antibiotic ciprofloxacin after LL-37 treatment. The response of *P. aeruginosa* against fluoroquinolone ciprofloxacin and its resistome has been extensively studied and reveals a complex regulation and an involvement of hundreds of different genes [52], [53], including RND efflux pumps MexAB-OprM, MexCD-OprJ and MexEF-OprN [3]. Since MexCD-OprJ was upregulated in reponse to LL-37 in our transcriptional analyses, the adaptive resistance against ciprofloxacin could be due, at least in parts, to this efflux pump induction. MexCD-OprJ efflux system is not expressed in wild-type *P. aeruginosa* under normal growth conditions, but can be initiated by mutations of repressor *nfxB.* In previous studies, this activation caused adaptive resistances to various substrates of MexCD-OprJ including macrolides, chloramphenicol, tetracycline and fluoroquinolones [54], [55] and led to a strain-specific induction of virulence [55]. It has been shown recently, that *P. aeruginosa mexCD-oprJ* expression is also stimulated by cationic biocides benzalkonium chloride and chlorhexidine gluconate [56], [57] and by other components causing membrane damage and envelope stress such as ethanol, SDS, polymyxin B and the antimicrobial peptides V8 and V681 in an *algU*-dependent pathway [57]. However, LL-37, although it is known to act as a cell membrane-damaging agent [58], did not cause alterations in *algU* sigma factor expression (see Tables S2 and S3) and the main regulator of MexCD-OprJ, the repressor *nfxB*, was rather upregulated than downregulated. These findings suggest the existence of an alternative peptide inducible regulator of MexCD-OprJ expression. Although previous experiments from other groups demonstrate that PqsE acts as positive regulator of *mexCD-oprJ* expression [20], [39], our qRT-PCR results indicate that other regulators may override the lack of PqsE in reponse to LL-37 leading to equally increased *mexD* levels in the PAO1-*pqsE* mutant as in the wild-type bacteria.

In addition to the increased ciprofloxacin resistance, susceptibility of *P. aeruginosa* to aminoglycoside gentamicin was also reduced following LL-37 treatment. Similar results have been reported for *Streptococcus pneumonaiae* and erythromycin [59]. Since aminoglycosides are mainly exported by the inducible efflux system MexXY-OprM [3], which was not affected by LL-37 in this study, the observed gentamicin resistance has to be caused by other factors. One possible explanation refers to the enhanced expression of the LPS modifying operon *arnBCDTEFugd* which mediates resistance to aminoglycosides and other cationic antibiotics [4]. In accord with a previous study [9], we observed a 1.9-fold increase in *pmrA* expression by LL-37, which is involved in *arn* regulation, whereas other two-component systems controlling *arn* expression, ParR-ParS [12], PhoP-PhoQ [10] and the recently identified system CprR-CprS [13], were not affected. Since *arnT* expression, but not *pmrA* expression, were stimulated in the PAO1-*pqsE* mutant by LL-37, the observed induction of *arnBCDTEFugd* expression seems to be independent of both, *pmrA* and *pqsE*.

Our observations that virulence factor production and adaptive resistance in response to LL-37, are in parts influenced by PqsE in a yet unknown manner emphasize the crucial role of quorum sensing in *P. aeruginosa* infections and the high potential of quorum sensing inhibitors as promising agents against infections caused by multi-resistant bacteria, as mentioned previously [60]. Since several cationic peptides, including LL-37, exhibit a potent antibiofilm activity, in several cases in combination with a direct antimicrobial activity against various bacterial species, their prospective medical application is widely discussed [15]. The possibility, that cationic compounds structurally related to LL-37 could be able to affect virulence and adaptive resistance in *P. aeruginosa* in a similar way, may limit their use as sole drug during infectious diseases and should be considered in further investigations.

## Supporting Information

Figure S1 ***P. aeruginosa*** **PAO1 cultures after 21 h of incubation with LL-37 (left) or without LL-37 (right) in MH medium under shaking conditions at 37°C.**
(TIF)

Table S1 qRT-PCR primers for detection of *P. aeruginosa* PAO1 gene expression.
(PDF)

Table S2 Full list of upregulated genes (fold change≥1.5) in response to 20 µg/ml LL-37 compared to non-treated *P. aeruginosa* PAO1.
(PDF)

Table S3 Full list of downregulated genes (fold change≥1.5) in response to 20 µg/ml LL-37 compared to non-treated *P. aeruginosa* PAO1.
(PDF)
