# Supplementary figures and images for: Human Host Defense Peptide LL-37 Stimulates Virulence Factor Production and Adaptive Resistance in Pseudomonas aeruginosa
Source: PLoS One. 2013 Dec 13;8(12):e82240. doi: 10.1371/journal.pone.0082240 (PMC3862677; doi:10.1371/journal.pone.0082240)

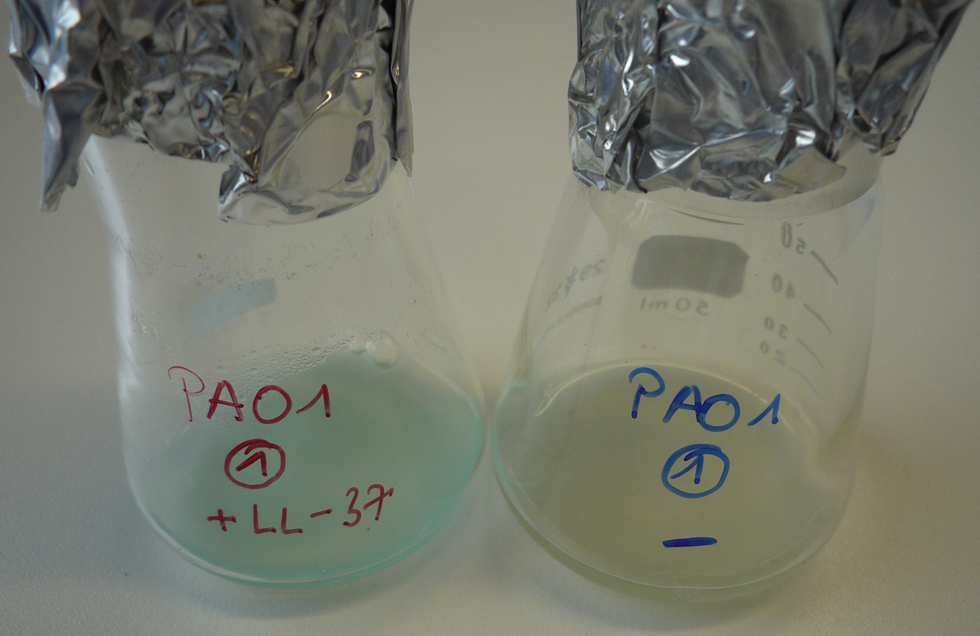

Supplement: Figure S1 — P. aeruginosa PAO1 cultures after 21 h of incubation with LL-37 (left) or without LL-37 (right) in MH medium under shaking conditions at 37°C. (TIF) [file pone.0082240.s001.tif]
