# Supplementary material for: Human Host Defense Peptide LL-37 Stimulates Virulence Factor Production and Adaptive Resistance in Pseudomonas aeruginosa
Source: PLoS One. 2013 Dec 13;8(12):e82240. doi: 10.1371/journal.pone.0082240 (PMC3862677; doi:10.1371/journal.pone.0082240)
Supplement: Table S1 — qRT-PCR primers for detection of P. aeruginosa PAO1 gene expression. (PDF) [file pone.0082240.s002.pdf]

Table S1: qRT-PCR primers for detection of *P. aeruginosa* PAO1 gene expression

PA number	Gene	Forward primer	Reverse primer
**PA4206**	*mexH*	TGG TGC AAC TCA ACG ACG C	GCA TGG AGG ATC TCG GCA TT
**PA1000**	*pqsE*	ATG ATG ACC TGT GCC TGT TG	GTC GTA GTG CTT GTG GGT GA
**PA4598**	*mexD*	ACC CGA AAT TCC TTT ACG CG	ATG GTC TCG AAG CTC ACC CC
**PA3724**	*lasB*	ATG AAC GAG GCG TTC TCC G	GTT GTA CAC GCC GCT GGA GT
**PA2194**	*hcnB*	AAC AAG CCG CAA CTG TTC CTC	TTC CGT CAG TTC CCC ATC G
**PA0576**	*rpoD*	GGG GAT CAA CGT ATT CGA GA	CAG TTC CAC GGT ACC CAT TT
**PA1901**	*phzC2*	GGA TCC TCA AGG GCT ATG C	GTG GGT CGA ACC GAG ATA GA
**PA3556**	*arnT*	ATG TGG TGC AGA ACA AGA CCC	CCG AGG TCA TTG CTC AGC A
**PA4776**	*pmrA*	AAC CAA CTG GAG CAG AGC CTC	ACG TGG ACT TCG ATG GCG T
**PA1430**	*lasR*	TGC CTA AGG ACA GCC AGG ACT	CCG AGG CTT CCT CGA AGA AC
**PA3477**	*rhlR*	TGC GTT GCA TGA TCG ATT G	CGC GTC GAA CTT CTT CTG GAT
