# Supplementary material for: Human Host Defense Peptide LL-37 Stimulates Virulence Factor Production and Adaptive Resistance in Pseudomonas aeruginosa
Source: PLoS One. 2013 Dec 13;8(12):e82240. doi: 10.1371/journal.pone.0082240 (PMC3862677; doi:10.1371/journal.pone.0082240)
Supplement: Table S2 — Full list of upregulated genes (fold change≥1.5) in response to 20 µg/ml LL-37 compared to non-treated P. aeruginosa PAO1. (PDF) [file pone.0082240.s003.pdf]

Table S2: Full list of upregulated genes (fold change ≥ 1.5) in response to 20 µg/ml LL-37 compared to non-treated *P. aeruginosa* PAO1

PA number	Gene name	Function class	Fold change
**PA0035**	*trpA*	Amino acid biosynthesis and metabolism	1.5
**PA0050**		Hypothetical, unclassified, unknown	2.5
**PA0051**	*phzH*	Putative enzymes	1.6
**PA0059**	*osmC*	Adaptation, Protection	1.5
**PA0062**		Hypothetical, unclassified, unknown	1.6
**PA0074**	*ppkA*	Adaptation, Protection; Translation, post-translational modification, degradation; Protein secretion/export apparatus	1.5
**PA0088**		Protein secretion/export apparatus	1.6
**PA0090**		Translation, post-translational modification, degradation; Chaperones & heat shock proteins; Protein secretion/export apparatus	1.9
**PA0091**		Protein secretion/export apparatus	1.9
**PA0098**		Hypothetical, unclassified, unknown	1.6
**PA0099**		Hypothetical, unclassified, unknown	1.6
**PA0102**		Central intermediary metabolism	2.0
**PA0103**		Membrane proteins; Transport of small molecules	2.0
**PA0122**		Adaptation, Protection	1.8
**PA0141**		Hypothetical, unclassified, unknown	1.5
**PA0169**		Motility & Attachment	1.8
**PA0175**		Transcriptional regulators; Adaptation, Protection; Chemotaxis	1.6
**PA0176**		Adaptation, Protection; Chemotaxis	1.5
**PA0177**		Adaptation, Protection; Chemotaxis	1.8

**PA0187**		Hypothetical, unclassified, unknown	2.6
**PA0269**		Hypothetical, unclassified, unknown	1.7
**PA0320**		Hypothetical, unclassified, unknown	7.2
**PA0327**		Hypothetical, unclassified, unknown	1.8
**PA0429**		Hypothetical, unclassified, unknown	1.6
**PA0430**	*metF*	Amino acid biosynthesis and metabolism; Central intermediary metabolism	2.0
**PA0431**		Hypothetical, unclassified, unknown	1.9
**PA0460**		Hypothetical, unclassified, unknown	1.5
**PA0506**		Putative enzymes	1.6
**PA0546**	*metK*	Amino acid biosynthesis and metabolism; Central intermediary metabolism	3.2
**PA0547**		Transcriptional regulators	3.1
**PA0589**		Energy metabolism	1.5
**PA0635**		Related to phage, transposon, or plasmid	1.5
**PA0744**		Putative enzymes	1.6
**PA0779**		Putative enzymes	1.9
**PA0793**		Hypothetical, unclassified, unknown	1.9
**PA0795**	*prpC*	Central intermediary metabolism; Carbon compound catabolism	1.9
**PA0796**	*prpB*	Fatty acid and phospholipid metabolism; Central intermediary metabolism; Carbon compound catabolism	2.5
**PA0852**	*cbpD*	Secreted Factors (toxins, enzymes, alginate)	1.6
**PA0853**		Putative enzymes	1.5
**PA0866**	*aroP2*	Transport of small molecules	1.7
**PA0887**	*acsA*	Carbon compound catabolism; Central intermediary metabolism	3.1
**PA0938**		Hypothetical, unclassified, unknown	1.5

**PA0996**	*pqsA*	Biosynthesis of cofactors, prosthetic groups and carriers	2.1
**PA0997**	*pqsB*	Biosynthesis of cofactors, prosthetic groups and carriers	1.9
**PA0998**	*pqsC*	Biosynthesis of cofactors, prosthetic groups and carriers	1.6
**PA0999**	*pqsD*	Biosynthesis of cofactors, prosthetic groups and carriers	1.7
**PA1000**	*pqsE*	Biosynthesis of cofactors, prosthetic groups and carriers	2.1
**PA1001**	*phnA*	Adaptation, Protection	2.0
**PA1002**	*phnB*	Amino acid biosynthesis and metabolism; Adaptation, Protection	2.2
**PA1070**	*braG*	Transport of small molecules	1.8
**PA1115**		Membrane proteins	2.2
**PA1119**		Membrane proteins; Cell wall / LPS / capsule	1.5
**PA1130**	*rhlC*	Cell wall / LPS / capsule; Adaptation, Protection	2.2
**PA1168**		Hypothetical, unclassified, unknown	8.0
**PA1169**		Putative enzymes	4.7
**PA1173**	*napB*	Energy metabolism	1.8
**PA1175**	*napD*	Energy metabolism	2.0
**PA1176**	*napF*	Energy metabolism	1.5
**PA1216**		Hypothetical, unclassified, unknown	2.0
**PA1217**		Amino acid biosynthesis and metabolism	1.8
**PA1323**		Hypothetical, unclassified, unknown	1.5
**PA1471**		Hypothetical, unclassified, unknown	1.7
**PA1478**		Hypothetical, unclassified, unknown	2.0
**PA1480**	*ccmF*	Energy metabolism	1.6
**PA1494**		Hypothetical, unclassified, unknown	1.5

**PA1550**		Hypothetical, unclassified, unknown	2.3
**PA1551**		Energy metabolism	2.0
**PA1556**		Energy metabolism; Energy metabolism	1.7
**PA1559**		Hypothetical, unclassified, unknown	7.9
**PA1560**		Hypothetical, unclassified, unknown	3.4
**PA1592**		Hypothetical, unclassified, unknown	1.5
**PA1657**		Hypothetical, unclassified, unknown	3.5
**PA1658**		Hypothetical, unclassified, unknown	2.8
**PA1659**		Hypothetical, unclassified, unknown	2.3
**PA1662**		Putative enzymes	1.7
**PA1664**		Hypothetical, unclassified, unknown	4.1
**PA1665**		Hypothetical, unclassified, unknown	2.3
**PA1666**		Hypothetical, unclassified, unknown	1.7
**PA1667**		Hypothetical, unclassified, unknown	2.3
**PA1668**		Hypothetical, unclassified, unknown	1.5
**PA1760**		Transcriptional regulators	1.5
**PA1797**		Hypothetical, unclassified, unknown	2.9
**PA1812**	*mltD*	Amino acid biosynthesis and metabolism; Cell wall / LPS / capsule	1.7
**PA1852**		Hypothetical, unclassified, unknown	2.0
**PA1869**		Fatty acid and phospholipid metabolism	2.5
**PA1871**	*lasA*	Secreted Factors (toxins, enzymes, alginate); Translation, post-translational modification, degradation	1.8
**PA1887**		Hypothetical, unclassified, unknown	1.5
**PA1888**		Hypothetical, unclassified, unknown	1.7
**PA1894**		Hypothetical, unclassified, unknown	1.6
**PA1895**		Membrane proteins	1.5

**PA1901**	*phzC2*	Secreted Factors (toxins, enzymes, alginate)	4.7
**PA1902**	*phzD2*	Secreted Factors (toxins, enzymes, alginate)	5.8
**PA1903**	*phzE2*	Secreted Factors (toxins, enzymes, alginate)	6.1
**PA1904**	*phzF2*	Secreted Factors (toxins, enzymes, alginate)	6.0
**PA1905**	*phzG2*	Secreted Factors (toxins, enzymes, alginate)	6.3
**PA1930**		Adaptation, Protection; Chemotaxis	1.5
**PA1969**		Hypothetical, unclassified, unknown	1.6
**PA2003**	*bdhA*	Carbon compound catabolism	1.7
**PA2011**	*gnyL*	Carbon compound catabolism	1.6
**PA2012**	*gnyA*	Carbon compound catabolism	3.5
**PA2013**	*gnyH*	Carbon compound catabolism	4.0
**PA2014**	*gnyB*	Carbon compound catabolism	3.8
**PA2015**	*gnyD*	Carbon compound catabolism	3.1
**PA2016**	*gnyR*	Transcriptional regulators	3.6
**PA2030**		Hypothetical, unclassified, unknown	2.5
**PA2031**		Hypothetical, unclassified, unknown	2.4
**PA2066**		Hypothetical, unclassified, unknown	2.1
**PA2067**		Putative enzymes	2.3
**PA2068**		Membrane proteins; Transport of small molecules	6.1
**PA2069**		Putative enzymes	6.8
**PA2109**		Hypothetical, unclassified, unknown	1.7
**PA2110**		Hypothetical, unclassified, unknown	1.6
**PA2112**		Hypothetical, unclassified, unknown	1.7
**PA2166**		Hypothetical, unclassified, unknown	1.7
**PA2171**		Hypothetical, unclassified, unknown	2.0
**PA2193**	*hcnA*	Central intermediary metabolism	2.4

**PA2194**	*hcnB*	Central intermediary metabolism	2.6
**PA2195**	*hcnC*	Central intermediary metabolism	2.1
**PA2274**		Hypothetical, unclassified, unknown	1.9
**PA2290**	*gcd*	Carbon compound catabolism; Energy metabolism	1.6
**PA2300**	*chiC*	Carbon compound catabolism	5.0
**PA2302**		Secreted Factors (toxins, enzymes, alginate); Putative enzymes	1.7
**PA2303**		Secreted Factors (toxins, enzymes, alginate)	2.0
**PA2358**		Hypothetical, unclassified, unknown	5.2
**PA2366**		Hypothetical, unclassified, unknown	2.1
**PA2367**		Hypothetical, unclassified, unknown	2.1
**PA2368**		Hypothetical, unclassified, unknown	2.3
**PA2433**		Hypothetical, unclassified, unknown	2.3
**PA2441**		Hypothetical, unclassified, unknown	1.6
**PA2485**		Hypothetical, unclassified, unknown	1.6
**PA2486**		Hypothetical, unclassified, unknown	1.7
**PA2552**		Putative enzymes	1.9
**PA2553**		Putative enzymes	1.9
**PA2554**		Putative enzymes	2.3
**PA2555**		Putative enzymes	1.6
**PA2557**		Fatty acid and phospholipid metabolism	3.0
**PA2562**		Hypothetical, unclassified, unknown	1.9
**PA2566**		Hypothetical, unclassified, unknown	2.0
**PA2570**	*pa1L*	Adaptation, Protection; Motility & Attachment; Cell wall / LPS / capsule	1.6
**PA2572**		Transcriptional regulators; Two-component regulatory systems	1.5
**PA2588**		Transcriptional regulators	1.5

**PA2607**		Hypothetical, unclassified, unknown	1.5
**PA2642**	*nuoG*	Energy metabolism	1.6
**PA2643**	*nuoH*	Energy metabolism	1.6
**PA2646**	*nuoK*	Energy metabolism	1.5
**PA2647**	*nuoL*	Energy metabolism	1.6
**PA2655**		Hypothetical, unclassified, unknown	1.6
**PA2717**	*cpo*	Central intermediary metabolism	1.5
**PA2754**		Hypothetical, unclassified, unknown	1.7
**PA2788**		Adaptation, Protection; Chemotaxis	2.1
**PA2895**		Hypothetical, unclassified, unknown	1.6
**PA3028**	*moeA2*	Biosynthesis of cofactors, prosthetic groups and carriers	1.5
**PA3038**		Transport of small molecules	2.2
**PA3049**	*rmf*	Translation, post-translational modification, degradation	1.7
**PA3126**	*ibpA*	Chaperones & heat shock proteins	2.1
**PA3149**	*wbpH*	Putative enzymes; Cell wall / LPS / capsule	1.6
**PA3213**		Hypothetical, unclassified, unknown	1.6
**PA3214**		Hypothetical, unclassified, unknown	1.5
**PA3234**		Membrane proteins; Transport of small molecules	1.8
**PA3235**		Membrane proteins	1.6
**PA3361**	*lecB*	Motility & Attachment	5.9
**PA3478**	*rhlB*	Secreted Factors (toxins, enzymes, alginate)	2.0
**PA3479**	*rhlA*	Secreted Factors (toxins, enzymes, alginate)	1.5
**PA3520**		Hypothetical, unclassified, unknown	1.6
**PA3530**		Hypothetical, unclassified, unknown	1.9
**PA3552**	*arnB*	Cell wall / LPS / capsule; Antibiotic resistance and susceptibility; Adaptation, Protection	1.6

**PA3553**	*arnC*	Adaptation, Protection; Putative enzymes; Cell wall / LPS / capsule; Antibiotic resistance and susceptibility	1.6
**PA3555**	*arnD*	Adaptation, Protection; Antibiotic resistance and susceptibility; Cell wall / LPS / capsule	1.5
**PA3556**	*arnT*	Adaptation, Protection; Membrane proteins; Cell wall / LPS / capsule	2.0
**PA3557**	*arnE*	Adaptation, Protection; Cell wall / LPS / capsule; Membrane proteins	2.0
**PA3558**	*arnF*	Membrane proteins; Adaptation, Protection; Cell wall / LPS / capsule	2.2
**PA3559**	*ugd*	Putative enzymes	2.8
**PA3569**	*mmsB*	Carbon compound catabolism	2.3
**PA3570**	*mmsA*	Amino acid biosynthesis and metabolism; Carbon compound catabolism	2.6
**PA3642**	*rnhB*	DNA replication, recombination, modification and repair	1.5
**PA3643**	*lpxB*	Cell wall / LPS / capsule	1.5
**PA3659**		Putative enzymes	1.6
**PA3661**		Hypothetical, unclassified, unknown	3.5
**PA3676**		Membrane proteins; Transport of small molecules	1.6
**PA3677**		Transport of small molecules	1.7
**PA3678**		Transcriptional regulators	1.7
**PA3719**		Antibiotic resistance and susceptibility	2.0
**PA3720**		Hypothetical, unclassified, unknown	2.6
**PA3724**	*lasB*	Secreted Factors (toxins, enzymes, alginate); Translation, post-translational modification, degradation	2.1
**PA3733**		Hypothetical, unclassified, unknown	1.5
**PA3734**		Hypothetical, unclassified, unknown	1.8
**PA3784**		Hypothetical, unclassified, unknown	2.3

**PA3785**		Hypothetical, unclassified, unknown	2.5
**PA3786**		Hypothetical, unclassified, unknown	1.9
**PA3809**	*fdx2*	Energy metabolism	1.6
**PA3810**	*hscA*	Chaperones & heat shock proteins	1.5
**PA3901**	*fecA*	Membrane proteins; Transport of small molecules	2.4
**PA3905**		Hypothetical, unclassified, unknown	2.0
**PA3906**		Hypothetical, unclassified, unknown	2.1
**PA3907**		Hypothetical, unclassified, unknown	1.7
**PA3908**		Hypothetical, unclassified, unknown	1.9
**PA3919**		Hypothetical, unclassified, unknown	1.7
**PA3981**		Hypothetical, unclassified, unknown	1.8
**PA3982**		Hypothetical, unclassified, unknown	1.6
**PA3996**	*lis*	Biosynthesis of cofactors, prosthetic groups and carriers	2.0
**PA4067**	*oprG*	Membrane proteins	1.6
**PA4129**		Hypothetical, unclassified, unknown	3.1
**PA4130**		Central intermediary metabolism	2.3
**PA4131**		Putative enzymes	2.5
**PA4132**		Hypothetical, unclassified, unknown	2.9
**PA4133**		Energy metabolism	3.7
**PA4134**		Hypothetical, unclassified, unknown	5.4
**PA4139**		Hypothetical, unclassified, unknown	9.6
**PA4140**		Hypothetical, unclassified, unknown	2.6
**PA4141**		Hypothetical, unclassified, unknown	3.2
**PA4142**		Protein secretion/export apparatus	1.5
**PA4205**	*mexG*	Membrane proteins	10.2
**PA4206**	*mexH*	Transport of small molecules	4.9

**PA4207**	*mexI*	Membrane proteins; Transport of small molecules	2.5
**PA4208**	*opmD*	Membrane proteins; Transport of small molecules	3.1
**PA4209**	*phzM*	Putative enzymes	5.3
**PA4210**	*phzA1*	Secreted Factors (toxins, enzymes, alginate)	7.8
**PA4211**	*phzB1*	Secreted Factors (toxins, enzymes, alginate)	4.5
**PA4217**	*phzS*	Putative enzymes	5.3
**PA4221**	*fptA*	Transport of small molecules	1.7
**PA4228**	*pchD*	Secreted Factors (toxins, enzymes, alginate); Transport of small molecules	1.6
**PA4229**	*pchC*	Secreted Factors (toxins, enzymes, alginate); Transport of small molecules	1.7
**PA4230**	*pchB*	Secreted Factors (toxins, enzymes, alginate); Transport of small molecules	1.8
**PA4231**	*pchA*	Secreted Factors (toxins, enzymes, alginate); Transport of small molecules	1.5
**PA4290**		Adaptation, Protection; Chemotaxis	1.6
**PA4306**		Motility & Attachment	2.0
**PA4359**		Hypothetical, unclassified, unknown	1.6
**PA4428**	*sspA*	Adaptation, Protection	1.6
**PA4430**		Energy metabolism	1.5
**PA4489**		Hypothetical, unclassified, unknown	2.0
**PA4490**		Hypothetical, unclassified, unknown	1.5
**PA4515**		Hypothetical, unclassified, unknown	2.7
**PA4525**	*pilA*	Motility & Attachment	1.6
**PA4573**		Hypothetical, unclassified, unknown	1.5
**PA4597**	*oprJ*	Membrane proteins; Antibiotic resistance and susceptibility; Transport of small molecules	3.9
**PA4598**	*mexD*	Transport of small molecules; Membrane proteins; Antibiotic resistance and susceptibility	4.4

**PA4599**	*mexC*	Transport of small molecules; Antibiotic resistance and susceptibility	9.1
**PA4600**	*nfxB*	Transcriptional regulators	1.9
**PA4611**		Hypothetical, unclassified, unknown	1.6
**PA4624**		Cell wall / LPS / capsule	1.9
**PA4625**		Secreted Factors (toxins, enzymes, alginate); Cell wall / LPS / capsule	3.0
**PA4675**		Transport of small molecules	1.9
**PA4700**	*mrcB*	Cell wall / LPS / capsule	1.5
**PA4757**		Membrane proteins	1.8
**PA4758**	*carA*	Amino acid biosynthesis and metabolism; Nucleotide biosynthesis and metabolism	1.6
**PA4773**		Hypothetical, unclassified, unknown	4.9
**PA4774**		Hypothetical, unclassified, unknown	3.2
**PA4775**		Hypothetical, unclassified, unknown	2.2
**PA4776**	*pmrA*	Two-component regulatory systems	1.9
**PA4782**		Hypothetical, unclassified, unknown	1.5
**PA4865**	*ureA*	Central intermediary metabolism	2.3
**PA4925**		Hypothetical, unclassified, unknown	1.7
**PA4946**	*mutL*	DNA replication, recombination, modification and repair	1.7
**PA4953**	*motB*	Membrane proteins; Adaptation, Protection; Chemotaxis	1.5
**PA4961**		Membrane proteins	1.7
**PA4997**	*msbA*	Fatty acid and phospholipid metabolism; Cell wall / LPS / capsule; Transport of small molecules	1.7
**PA5070**	*tatC*	Membrane proteins; Transport of small molecules	1.6
**PA5182**		Membrane proteins	2.2
**PA5183**		Membrane proteins	2.7

**PA5217**		Transport of small molecules	2.0
**PA5220**		Hypothetical, unclassified, unknown	2.0
**PA5278**	*dapF*	Amino acid biosynthesis and metabolism	1.9
**PA5314**		Hypothetical, unclassified, unknown	1.6
**PA5323**	*argB*	Amino acid biosynthesis and metabolism	1.5
**PA5338**	*spoT*	Nucleotide biosynthesis and metabolism; Adaptation, Protection	1.5
**PA5339**		Hypothetical, unclassified, unknown	2.7
**PA5340**		Hypothetical, unclassified, unknown	1.6
**PA5362**		Hypothetical, unclassified, unknown	1.5
**PA5445**		Putative enzymes	1.9
**PA5473**		Membrane proteins	1.5
**PA5475**		Hypothetical, unclassified, unknown	1.7
**PA5496**		Nucleotide biosynthesis and metabolism	1.8
**PA5497**		Nucleotide biosynthesis and metabolism	1.9
**PA5526**		Hypothetical, unclassified, unknown	1.6
**PA5531**	*tonB*	Transport of small molecules	2.0
