# Supplementary material for: Human Host Defense Peptide LL-37 Stimulates Virulence Factor Production and Adaptive Resistance in Pseudomonas aeruginosa
Source: PLoS One. 2013 Dec 13;8(12):e82240. doi: 10.1371/journal.pone.0082240 (PMC3862677; doi:10.1371/journal.pone.0082240)
Supplement: Table S3 — Full list of downregulated genes (fold change≥1.5) in response to 20 µg/ml LL-37 compared to non-treated P. aeruginosa PAO1. (PDF) [file pone.0082240.s004.pdf]

Table S3: Full list of downregulated genes (fold change ≥ 1.5) in response to 20 μg/ml LL-37 compared to non-treated *P. aeruginosa* PAO1

PA number	Gene name	Function class	Fold change
**PA0026**	*plcB*	Hypothetical, unclassified, unknown	-1.9
**PA0115**		Hypothetical, unclassified, unknown	-1.5
**PA0277**		Hypothetical, unclassified, unknown	-1.7
**PA0347**	*glpQ*	Fatty acid and phospholipid metabolism	-3.6
**PA0450**		Membrane proteins; Transport of small molecules	-1.5
**PA0603**		Transport of small molecules	-2.2
**PA0604**		Transport of small molecules	-2.4
**PA0605**		Membrane proteins; Transport of small molecules	-2.6
**PA0606**		Membrane proteins; Transport of small molecules	-1.8
**PA0674**		Transcriptional regulators	-5.1
**PA0675**		Transcriptional regulators	-3.4
**PA0676**		Membrane proteins; Transcriptional regulators	-1.9
**PA0679**		Hypothetical, unclassified, unknown	-2.1
**PA0681**		Protein secretion/export apparatus	-2.3
**PA0682**		Protein secretion/export apparatus	-1.7
**PA0683**		Protein secretion/export apparatus	-1.5
**PA0685**		Protein secretion/export apparatus	-2.2
**PA0688**		Transport of small molecules	-2.8
**PA0691**		Hypothetical, unclassified, unknown	-6.0
**PA0692**		Hypothetical, unclassified, unknown	-3.0
**PA0693**	*exbB2*	Membrane proteins; Transport of small molecules	-6.4
**PA0694**	*exbD2*	Membrane proteins; Transport of small molecules	-2.2

**PA0696**		Hypothetical, unclassified, unknown	-3.6
**PA0697**		Hypothetical, unclassified, unknown	-2.4
**PA0698**		Hypothetical, unclassified, unknown	-2.6
**PA0730**		Putative enzymes	-2.3
**PA0750**	*ung*	DNA replication, recombination, modification and repair	-1.5
**PA0775**		Hypothetical, unclassified, unknown	-1.6
**PA0842**		Putative enzymes	-2.9
**PA0975**		Putative enzymes	-1.5
**PA0979**		Related to phage, transposon, or plasmid	-2.0
**PA1004**	*nadA*	Amino acid biosynthesis and metabolism; Biosynthesis of cofactors, prosthetic groups and carriers	-1.5
**PA1014**		Putative enzymes	-1.5
**PA1051**		Membrane proteins; Transport of small molecules	-3.3
**PA1052**		Hypothetical, unclassified, unknown	-1.7
**PA1078**	*flgC*	Cell wall / LPS / capsule; Motility & Attachment	-1.5
**PA1081**	*flgF*	Cell wall / LPS / capsule; Motility & Attachment	-1.5
**PA1082**	*flgG*	Cell wall / LPS / capsule; Motility & Attachment	-1.6
**PA1083**	*flgH*	Cell wall / LPS / capsule; Motility & Attachment	-1.5
**PA1116**		Hypothetical, unclassified, unknown	-2.5
**PA1134**		Hypothetical, unclassified, unknown	-1.7
**PA1190**		Membrane proteins	-2.8
**PA1225**		Putative enzymes	-1.5
**PA1299**		Hypothetical, unclassified, unknown	-1.5
**PA1325**		Hypothetical, unclassified, unknown	-1.5
**PA1326**	*ilvA2*	Amino acid biosynthesis and metabolism	-1.5
**PA1327**		Putative enzymes	-1.5

**PA1383**		Hypothetical, unclassified, unknown	-1.6
**PA1606**		Hypothetical, unclassified, unknown	-4.9
**PA1607**		Hypothetical, unclassified, unknown	-1.6
**PA1632**	*kdpF*	Transport of small molecules	-2.1
**PA1633**	*kdpA*	Transport of small molecules	-2.6
**PA1634**	*kdpB*	Transport of small molecules	-2.2
**PA1635**	*kdpC*	Transport of small molecules	-2.0
**PA1636**	*kdpD*	Two-component regulatory systems	-2.0
**PA1654**		Putative enzymes	-1.5
**PA1706**	*pcrV*	Protein secretion/export apparatus	-1.5
**PA1839**		Hypothetical, unclassified, unknown	-1.5
**PA1863**	*modA*	Transport of small molecules	-1.6
**PA1882**		Membrane proteins; Transport of small molecules	-1.6
**PA1913**		Hypothetical, unclassified, unknown	-1.6
**PA2022**		Putative enzymes	-2.7
**PA2203**		Membrane proteins; Transport of small molecules	-1.6
**PA2306**		Membrane proteins; Secreted Factors (toxins, enzymes, alginate)	-1.7
**PA2381**		Hypothetical, unclassified, unknown	-1.6
**PA2427**		Hypothetical, unclassified, unknown	-2.3
**PA2428**		Hypothetical, unclassified, unknown	-3.1
**PA2548**		Hypothetical, unclassified, unknown	-3.8
**PA2635**		Hypothetical, unclassified, unknown	-5.5
**PA2654**		Adaptation, Protection; Chemotaxis	-1.9
**PA2803**		Hypothetical, unclassified, unknown	-3.2
**PA2804**		Hypothetical, unclassified, unknown	-3.5
**PA2850**	*ohr*	Adaptation, Protection	-2.0

**PA2880**		Hypothetical, unclassified, unknown	-1.5
**PA2881**		Transcriptional regulators; Two-component regulatory systems	-4.8
**PA2882**		Two-component regulatory systems	-3.1
**PA3116**		Amino acid biosynthesis and metabolism	-1.6
**PA3120**	*leuD*	Amino acid biosynthesis and metabolism	-1.6
**PA3219**		Hypothetical, unclassified, unknown	-5.3
**PA3250**		Hypothetical, unclassified, unknown	-1.6
**PA3251**		Hypothetical, unclassified, unknown	-1.6
**PA3258**		Hypothetical, unclassified, unknown	-4.8
**PA3278**		Membrane proteins	-1.8
**PA3279**	*oprP*	Transport of small molecules	-5.5
**PA3280**	*oprO*	Transport of small molecules	-8.2
**PA3296**	*phoA*	Central intermediary metabolism	-6.7
**PA3319**	*plcN*	Secreted Factors (toxins, enzymes, alginate)	-5.9
**PA3368**		Putative enzymes	-2.8
**PA3371**		Hypothetical, unclassified, unknown	-1.6
**PA3374**		Transport of small molecules	-1.6
**PA3375**		Transport of small molecules	-2.0
**PA3376**		Transport of small molecules	-1.7
**PA3377**		Transport of small molecules	-2.3
**PA3378**		Transport of small molecules	-2.7
**PA3379**		Transport of small molecules	-2.6
**PA3380**		Transport of small molecules	-2.8
**PA3381**		Transcriptional regulators	-3.5
**PA3382**	*phnE*	Membrane proteins; Transport of small molecules	-8.3
**PA3383**		Transport of small molecules	-7.6

**PA3384**	*phnC*	Transport of small molecules	-7.9
**PA3432**		Membrane proteins	-1.6
**PA3436**		Hypothetical, unclassified, unknown	-1.9
**PA3443**		Membrane proteins; Transport of small molecules	-1.6
**PA3531**	*bfrB*	Transport of small molecules; Adaptation, Protection	-1.9
**PA3841**	*exoS*	Secreted Factors (toxins, enzymes, alginate)	-1.6
**PA3909**		Hypothetical, unclassified, unknown	-3.6
**PA3910**		Nucleotide biosynthesis and metabolism; Secreted Factors (toxins, enzymes, alginate)	-7.1
**PA3925**		Putative enzymes	-1.5
**PA3990**		Hypothetical, unclassified, unknown	-3.0
**PA4138**	*tyrS*	Amino acid biosynthesis and metabolism; Translation, post-translational modification, degradation	-2.0
**PA4307**	*pctC*	Adaptation, Protection; Chemotaxis	-2.0
**PA4335**		Hypothetical, unclassified, unknown	-1.6
**PA4350**		Fatty acid and phospholipid metabolism	-8.6
**PA4351**		Fatty acid and phospholipid metabolism; Fatty acid and phospholipid metabolism	-10.4
**PA4443**	*cysD*	Central intermediary metabolism; Amino acid biosynthesis and metabolism	-1.5
**PA4582**		Hypothetical, unclassified, unknown	-1.9
**PA4583**		Hypothetical, unclassified, unknown	-2.1
**PA4635**		Hypothetical, unclassified, unknown	-2.4
**PA4656**		Hypothetical, unclassified, unknown	-1.7
**PA4658**		Hypothetical, unclassified, unknown	-1.5
**PA4821**		Membrane proteins; Transport of small molecules	-2.2
**PA4822**		Membrane proteins	-1.6

**PA4823**		Hypothetical, unclassified, unknown	-1.5
**PA4826**		Hypothetical, unclassified, unknown	-1.5
**PA4844**		Adaptation, Protection; Chemotaxis	-4.2
**PA4985**		Hypothetical, unclassified, unknown	-1.6
**PA5082**		Transport of small molecules	-2.0
**PA5297**	*poxB*	Central intermediary metabolism; Energy metabolism	-1.7
**PA5360**	*phoB*	Transcriptional regulators; Two-component regulatory systems	-2.6
**PA5361**	*phoR*	Two-component regulatory systems	-1.7
**PA5365**	*phoU*	Membrane proteins; Transcriptional regulators	-1.6
**PA5366**	*pstB*	Membrane proteins; Transport of small molecules	-1.8
**PA5367**	*pstA*	Membrane proteins; Transport of small molecules	-2.2
**PA5368**	*pstC*	Membrane proteins; Transport of small molecules	-2.0
**PA5369**		Transport of small molecules	-1.9
**PA5389**		Transcriptional regulators	-1.5
**PA5436**		Central intermediary metabolism	-1.6
